# Vaccum drainage system application in the management of operation-related non-regional epidural hematoma

**DOI:** 10.1186/1750-1164-7-7

**Published:** 2013-07-10

**Authors:** Jun Ma, Huan Li, Linggang Cheng, Song Lin

**Affiliations:** 1 Department of Neurosurgery, Capital Medical University-Beijing Tiantan Hospital, Beijing, China; 2 Beijing Tiantan Hospital, Dongcheng District, Beijing, China

**Keywords:** Intracranial tumor, Operation-related epidural hematoma, Non-regional epidural hematoma, Epidural drainage, Vaccum drainage system

## Abstract

**Background:**

Epidural intracranial hematoma is one of the most common complications of surgeries for intracranial tumors. The non-regional epidural hematoma is related to severe fluctuation of the intracranial pressure during the operation. The traditional management of hematoma evacuation through craniotomy is time-consuming and may aggravate intracranial pressure imbalance, which causes further complications. We designed a method using vaccum epidural drainage system, and tried to evaluate advantage and the disadvantage of this new technique.

**Methods:**

Seven patients of intracranial tumors were selected. All of the patients received tumor resection and intra-operative non-regional epidural hematoma was confirmed through intra-operative ultrasound or CT scan. The vaccum drainage system was applied. Another ten patients who received craniotomy for intra-operative non-regional epidural hematoma evacuation were selected as comparison. Regular tests, like serial CT scan, were performed afterward to evaluate the effectiveness and to help deciding when to remove the drainage system.

**Results:**

The vaccum drainage method was effective in epidual hemotoma clearance and prevented recurrent epidural hemorrhage. The drainage systems were removed within 4 days. All of the patients recovered well. No complications related to the drainage system were observed.

**Conclusions:**

Compared to the traditional craniotomy, the new method of epidural hemoatoma management using vaccum epidural drainage system proved to be as effective in hematoma clearance, and was less-invasive and easier to perform, with less complication, shorter hospitalization, less economic burden, and better prognosis.

## Background

Epidural intracranial hematoma (EDH) is one of the most common complications of surgeries of intracranial tumors. The incidence is 1.4%, among the three most frequent reasons of re-operation during the early time (with the other two as brain edema and hematoma in the empty space left by lesion evacuation). Intra-operative non-regional (adjacent or distant) EDH is an emergency and may cause abnormal performances like protrusion of the cortex through the bone flap or especially difficult coagulation, although the signs are not always revealing. Hematoma evacuation through craniotomy is the traditional management. However the re-operation can cause further complications, like recurrent regional EDH, regional cerebral hematoma, contra-lateral distant EDH, and contra-lateral distant cerebral hematoma, etc. Sometimes the patient cannot afford to the consecutive strikes, and the mortality of re-operation comes up to approximately 12%. Actually the situation is always so urgent that the preparation of re-operation may delay the rescue. As a well-known but poorly understood complication, operation-related EDH presents to be a great challenge of peri-operative recovery. A fast, less-invasive method which is also effective and practicable, with a low rate of mortality is highly required. The EDH formation is mainly ascribed to the rapid fall of the intracranial pressure, which may be related to a series of conditions, like the size of the tumor, the extent of excision, and the fluctuation of blood pressure, etc. The falling down of the intracranial pressure tears up the potential epidural space and switches on the evil circle of epidural hemorrhage. On the basis of these hypotheses, we designed a method to control EDH using continuous vaccum epidural drainage, intending to improve the overall prognosis. The method proved to be effective with little complication, and the patients recovered soon.

## Methods

We gather the information of in-patients with intracranial tumors in the neurosurgery department of our hospital since Oct. 2011. Each of the patients undertook craniotomy and developed non-regional EDH as an intra-operative complication. The patients with regional EDH, of emergency, with significant pre-operative accompanying diseases, or who were operated on for lesions other than intracranial tumors were excluded. According to the approach of the initial operation, operation-related EDH was classified as regional hematoma and non-regional hematoma, thus developed at the adjacent or distant epidural space. The regional EDH is often caused by inappropriate maneuver, and is beyond this investigation.

7 recent cases were included; all patients were operated on for intracranial tumors, and developed intra-operative non-regional EDH after or during the tumor resection. The phenomena of abnormal protrusion of the cortex, continuous epidural hemorrhage, or difficult coagulation during the operation were highly suggestive, and BUS or CT scan would confirm the situation immediately. A new “drainage system” was applied on these 7 patients, thus evacuated the hematoma by aspiration through the epidural space for adjacent EDH or through a burr hole for distant EDH, and applied constant vacuum aspiration in the epidural space. Sometimes the EDH is adjacent to the bone flap, and reliable coagulation could be achieved for active hemorrhage through the epidural space directly. For non-arterial hemorrhage compression hemostasis with absorbable gelatin sponge would be effective. To achieve sufficient and effective vaccum drainage, the drainage catheter was made of a couple of lateral holes on the terminal edge, and these lateral holes must be inserted within the epidural space completely. The peripheral dura, which would be split away from the inner periosteum widely during hematoma clearance, had to be suspended tightly for adjacent EDH. The residual blood was irrigated thoroughly before a continuous vaccum suction device was attached to the other end of the catheter. As all EDH was confirmed by intra-operative ultrasound (BUS) or CT-scan for these 7 patients and the application of hematoma drainage system was performed immediately, we chose the another 10 patients before Oct. 2011 with operation-related non-regional EDH who were re-operated on within the same day of the EDH development as control group in order that the results were more comparative, although the time of EDH development varied. These 10 cases represented most typical complications of re-operation. All EDH were confirmed by CT scan.

The 7 patients with vacuum drainage system application were monitored for critical signs, CBC, hemostasis, drainage volume, and CT performance regularly. All 17 patients were documented for further complications, the time of hospitalization, and the states of discharging.

This vacuum drainage method has been approved by each patient and the informed consent form has been signed before the operation. This study is approved by Institutional Review Board of Beijing Tiantan Hospital Affiliated to Capital Medical University.

## Results

Of all 17 included cases, 9 were male & 8 were female, averaging 35 years old, ranging from 1 to 57 years old. The original tumors involved 4 meningioma, 3 haemangiopericytoma, 2 ependymoma, 2 Schwannoma, 1 glioma, 1 haemangioblastoma, 1 teratoma, 1 craniopharygioma, 1 centroneurocytoma, and 1 epidermoid cyst. Most were large tumors with severe compression effect, like prominent median line shift or hydrocephalus, etc. The 3 points fixtor was applied to all patients during the operation of tumor resection. Of 10 patients with EDH who were re-operated on, the time of EDH development ranged from 0 to 5 days, taking the day of the original operation as day 0. The patients developed EDH on day 0 were actually intra-operative EDH and were further operated on with craniotomy immediately (Tables 1 and 2).

**Table 1:** Patients with “drainage system” application for operation-related EDH confirmed by BUS or CT-scan

	**Patient**	**Tumor**		**Surgery**					**Hematoma**
**No.**	**Gender, Age (yr.)**	**Location**	**Diagnosis**	**Approach**	**Blood loss (ml)**	**BP (mmHg)**	**Income/Outcome (ml)**	**Period of operation**	**Location**
1	F, 42	left fronto-parietal lobe, parasaggital (type IV)	fibrous meningioma	right lateral supine position, left fronto-parietal approach	3600	102/63	9920/6600	6 hr. 18 min.	right fronto-temporal-parietal
2	M, 28	retro-III^rd^ ventricle	epidermoid cyst	left lateral supine position, right POPPEN approach	2500	121/77	6570/4500	6 hr. 25 min.	left temporal-occipital
3	F, 44	left parietal, parasaggital (type IV)	meningothelial meningioma	right lateral supine position, left parietal approach	600	110/64	3850/3100	3 hr. 43 min.	right parietal
4	M, 50	tentorial (incisurial)	anaplastic haemangiopericytoma	left lateral supine position, right POPPEN approach	4000	123/82	8290/6500	7 hr. 16 min.	left temporal-occipital
5	F, 41	bi-parietal, parasaggital (type V)	transitional meningioma	supine position, bi-parietal approach	600	93/59	5100/2600	4 hr. 21 min.	right temporal-parietal
6	M, 26	sellar region	craniopharygioma	supine position, right fronto-temporal approach	400	112/70	3600/2400	3 hr. 20 min.	left fronto-temporal
7	F, 23	bi-lateral ventricle	centroneurocytoma	supine position, left trans-callosal approach	400	116/65	4400/2600	4 hr. 10 min	right parietal

**Table 2:** Patients with hematoma evacuation of craniotomy for operation-related EDH confirmed by CT-scan

	**Patient**	**Tumor**		**Surgery**					**Hematoma**	
**No.**	**Gender, Age (yr.)**	**Location**	**Diagnosis**	**Approach**	**Blood loss (ml)**	**BP (mmHg)**	**Income/Outcome (ml)**	**Period of operation**	**Location**	Time of appearance*
1	M, 1	left parietal-occipital, basal ganglia	ependymoma	left parietal-occipital	100	110/65	2200/1500	4 hr. 53 min.	left frontal	Day 1
2	F, 38	left frontal	oligo-astrocytoma	left frontal	2500	134/88	9880/6500	9 hr. 20 min.	right frontal	Day 0
3	F, 32	cerebellar vermis, brain stem	haemangioblastoma	posterior central	1800	113/74	7050/3800	7 hr. 8 min.	left temporal-parietal	Day 1
4	M, 57	C1-6	recurrent haemangiopericytoma	posterior central	300	118/83	3000/1800	4 hr. 44 min.	right fronto-temporal-parietal	Day 6
5	M, 31	left CPA	Schwannoma	left CPA	1000	125/75	5750/3100	11 hr.	left temporal-parietal	Day 0
6	M, 19	the IV^th^ ventricle	ependymoma	posterior central	350	127/70	3000/1850	5 hr. 17 min.	right cerebellar-and-occipital	Day 2
7	M, 39	right jugular vein foreman	Schwannoma	right posterior paracentral	500	154/89	3900/2000	6 hr. 19 min.	bilateral occipital	Day 5
8	M, 42	posterior fossa	haemangiopericytoma	posterior central	5500	114/77	10370/8000	10 hr. 59 min.	left temporal-parietal-occipital	Day 1
9	F, 32	left frontal	fibroblastic meningioma	left frontal-temporal	1000	116/79	4750/3000	7 hr. 47 min.	left temporal-parietal	Day 0
10	M, 17	trigonal area of left lateral ventricle	mature teratoma	left temporal-occipital	1500	120/70	6150/4000	10 hr. 33 min.	bilateral frontal	Day 0

*Take the day of the original operation as day 0.

**Figure 1:**
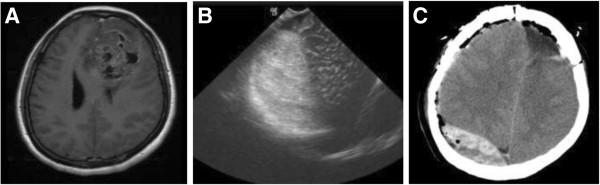
**Case 2 from the re-operation group.** The patient operated on for left frontal oligo-astrocytoma developed right frontal EDH. Re-operation of EDH evacuation through craniotomy were performed, with further formation of contralateral EDH after the operation. **A**: Pre-op. MR of patient 2 (transverse, enhanced T1-weighted), showing tumor in the left frontal lobe, with heterogeneous T1-weighted signal and heterogeneous enhancement. The tumor was confirmed as oligo-astrocytoma by postoperative pathological examination. **B**: Intraoperative BUS identifying right frontal EDH. The patient was operated on through left frontal approach. **C**: CT scan 1 day after the right frontal epidural hematoma evacuation, identifying right parietal-occipital epidural hematoma.

**Figure 2:**
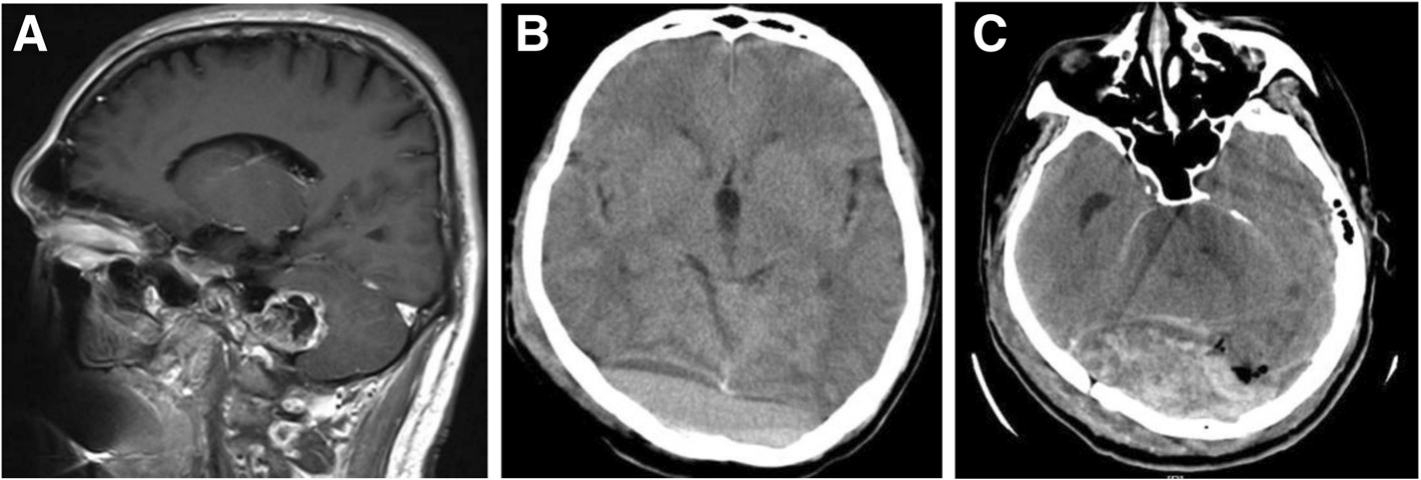
**Case 7 from the re-operation group.** The patient operated on for infratentorial Schwannoma developed supratentorial EDH. Re-operation of EDH evacuation through craniotomy were performed, with further formation of recurrent EDH after the operation. **A**: Pre-op. MR of patient 7 (saggital, enhanced T1-weighted), showing tumor in the right jugular foramen, with irregular heterogeneous enhancement. The tumor was confirmd as Schwannoma by postoperative pathological examination. **B**: Post-op. CT scan 5 days after the operation, identifying bilateral occipital EDH. The patient was operated on through right posterior paracentral approach. Bilateral occipital epidural hyperintensive signal could be seen. **C**: CT scan 7 days after the bilateral occipital epidural hematoma evacuation, identifying recurrent epidural hematoma of the same location.

**Figure 3:**
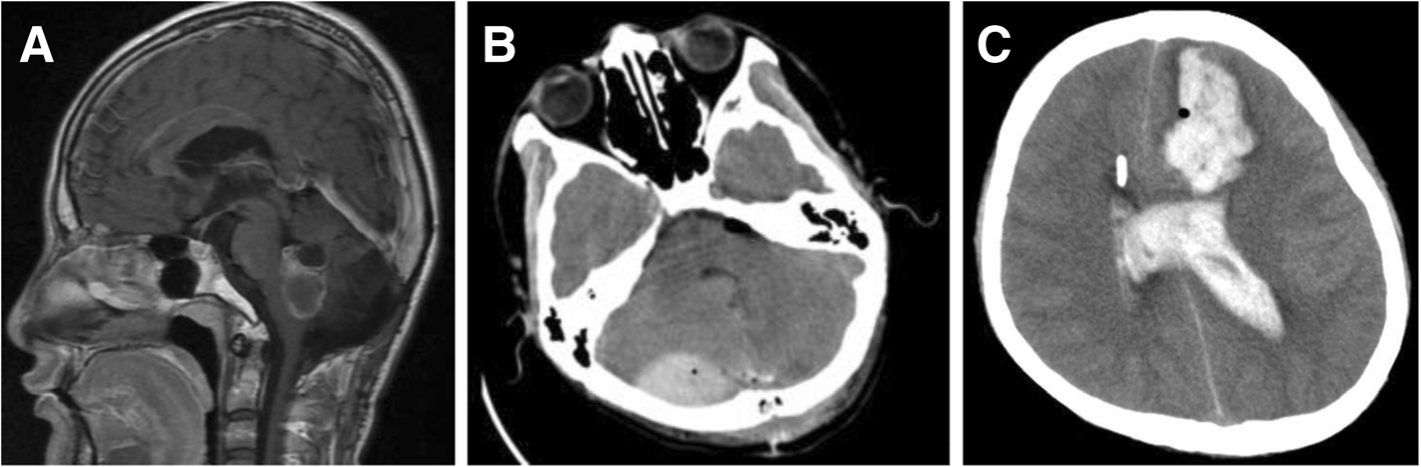
**Case 6 from the re-operation group.** The patient operated on for infratentorial ependymoma developed infratentorial-supratentorial EDH. Re-operation of EDH evacuation through craniotomy were performed, with further formation of contralateral cerebral hematoma after the operation. **A**: Pre-op. MR of case 6 (saggital, enhanced T1-weighted), showing tumor in the IV^th^ ventricle, with heterogeneous enhancement and clear margin. The patient had concomitant hydrocephalus. The tumor was confirmed as ependymoma by postoperative pathological examination. **B**: Post-op. CT scan 2 days after the operation, identifying right cerebellar and right occipital EDH. The patient was operated on through posterior central approach. Right occipital epidural hyperintensive signal could be seen. Consecutive images showed the hematoma ranged from infratentorial to supratentorial epidural space. **C**: CT scan 5 days after the right cerebellar-and-occipital epidural hematoma evacuation, identifying left frontal cerebral hematoma. The hemorrhage drained into lateral ventricles.

In the group of re-operation with craniotomy, 2 recovered with no further complications, 4 received further re-operation due to contra-lateral cerebral hematoma, recurrent regional EDH, regional cerebral hematoma, and malignant intracranial hypertension, respectively. 2 with further contra-lateral distant EDH and contra-lateral cerebral hematoma received conservative treatment. And another 2 patients did not survive (Table 3) (Figures 1, 2 and 3).

**Table 3:** Follow-up management and prognosis of patients with re-operation of craniotomy on the day of EDH development

**No.**	Postoperative management-hematoma evacuation with craniotomy*	**Prognosis**
1	evacuation of left frontal EDH	better, discharged 26/25 days after the 1^st^/2^nd^ operation
2	evacuation of right frontal EDH	right parietal-occipital EDH development 1 day later with conservative treatment
3	evacuation of left temporal-parietal EDH	other**
4	evacuation of right fronto-temporal-parietal EDH	dead 1 day later
5	evacuation of left temporal-parietal EDH	better, discharged 15 days after operation
6	evacuation of right occipital EDH	re-operation of left frontal cerebral hematoma 5 days later
7	evacuation of bilateral occipital EDH	re-operation of recurrent bilateral occipital EDH 7 days later
8	evacuation of left temporal-parietal-occipital EDH	re-operation of left temporal-parietal-occipital cerebral hematoma 1 day later
9	evacuation of left temporal-parietal EDH	decompressive craniectomy 3 days later due to left cerebral edema with serious intracranial hypertension
10	evacuation of bilateral frontal EDH	left parietal cerebral hemorrhage development 1 day later with conservative treatment

*All patients were operated on the day of EDH appearance.

**The patient gave up treatment with poor prognosis.

**Figure 4:**
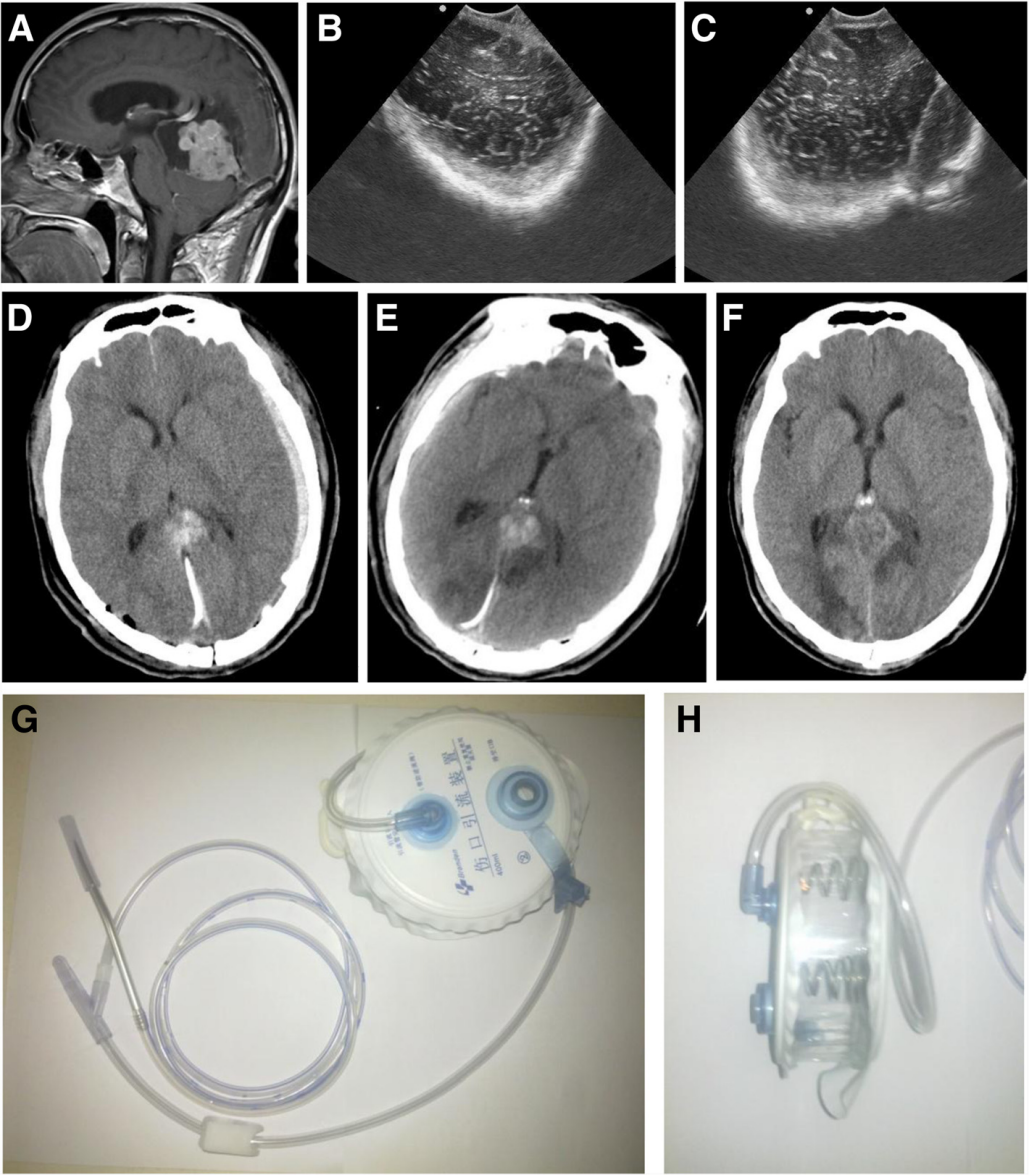
**Case 4 from the drainage group.** The patient operated on for the III^rd^ ventricle anaplastic haemangiopericytoma developed left temporal-occipital EDH. Epidural drainage with the vacuum system was performed. The drainage was removed 2 days later, with no further complications. **A**: Pre-op. MR of patient 4 (saggital, enhanced T1-weighted), showing tumor in the III^rd^ ventricle, with heterogeneous prominent enhancement. The patient has accompanying hydrocephalus. The tumor was confirmed as anaplastic haemangiopericytoma by postoperative pathological examination. **B** &**C**: Intraoperative BUS identifying left temporal-occipital EDH after tumor resection. The patient was operated on through right POPPEN approach. **D**: Post-op. CT scan a couple of hours after the operation. Left epidural hyperintensive signal and the draining cathether could be seen. There was also another catheter draining the residual cavity left by tumor resection. **E**: Post-op. CT scan before epidural drainage removal 2 days later. The epidural hyperintensive signal reduced prominently. **F**: CT scan of the patient after discharging. There was no abnormal signal of the epidural space. **G** &**H**: The vacuum drainage system. The vacuum was formed through the strength of the springs, and the volume capacity is 400 ml.

**Figure 5:**
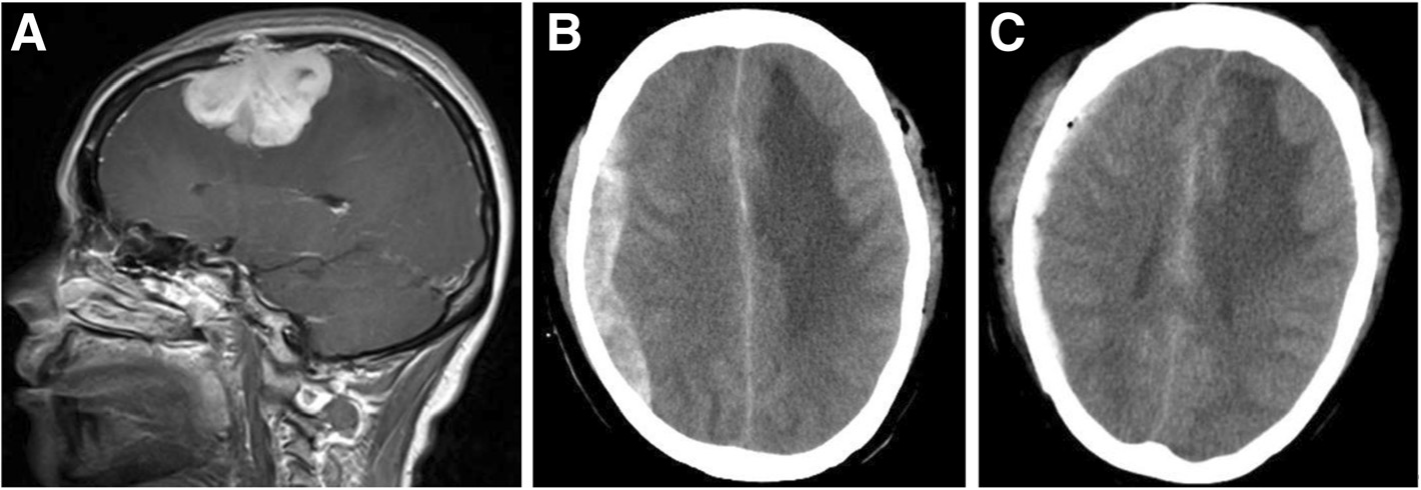
**Case 5 from the drainage group.** The patient operated on for bi-parietal meningioma developed right temporal-parietal EDH. Epidural drainage with the vacuum system was performed. The drainage was removed 1 day later, with no further complications. **A**: Pre-op. MR of patient 5 (saggital, enhanced T1-weighted), showing tumor of the bi-parietal lobes, with prominent enhancement and adjacent meninges enhancement. The tumor was confirmed as transitional meningioma by postoperative pathological examination. **B**: Postoperative CT scan identifying right temporal-parietal EDH after tumor resection. The patient was operated on through bi-parietal approach. **C**: Post-op. CT scan before epidural drainage removal 1 day later. The epidural hyperintensive signal reduced prominently.

In the group of drainage system, the patients recovered uneventfully. With supportive treatment he critical signs, the CBC and hemostasis results, etc. were relatively stable. The vacuum drainage catheter was removed when the hemorrhage volume was less than 100 ml per day, and the CT-scan was negative showing no obvious epidural hyper-intensive signal. The drainage system was maintained for 1 day for 1, 2 days for 4, and 4 days for 2. 5 patients were discharged walking independently, with trivial symptoms including heminopia and hemiparesis. 1 patient with left fronto-parietal para-saggital meningioma got right-side hemiplegia and went on for rehabilitation, and so is 1 patient with centroneurocytoma of bi-lateral ventricle. The symptoms were not considered EDH-related. The follow-up of 4 to 7 months showed no recurrent EDH or other complications (Table 4) (Figures 4 and 5).

**Table 4:** Follow-up management and prognosis of patients with drainage system application

**No.**	**Postoperative management-continuous drainage and than remove**	**Prognosis**
1	remove drainage 2 days later	better, discharged 9 days after operation
2	remove drainage 4 days later	go on for rehabilitation treatment
3	remove drainage 1 day later	better, discharged 28 days after operation
4	remove drainage 2 days later	better, discharged 6 days after operation
5	remove drainage 2 days later	better, discharged 8 days after operation
6	remove drainage 2 days later	better, discharged 10 days after operation
7	remove drainage 4 days later	go on for rehabilitation treatment

## Discussion

The epidural space is a potential space, which normally does not exist. Meningeal veins, bridging veins, and meningeal arteries puncture the dura mater. In some circumstances, a torn blood vessel may cause hemorrhage which is sufficient to split the space between the 2 dural layers and create epidural space, forming an epidural hematoma. Deduced from 17 cases of this study, the potential epidural space is split mainly by forceful suction from the outer side, formed by a rapid or significant fall of intracranial pressure (ICP), accompanied by disaster hemorrhage into the split space with insufficient self-coagulation, consistent with widely-accepted hypothesis. The incidence of operation-related EDH is relatively higher for large tumor, with total excision, or rapid CSF releasing, and may also for those with longer lasting operations, especially cases with intra-operative fall of the blood pressure [1-4]. Operation-related EDH is an emergency, which would cause abnormal performances like protrusion of the cortex through a bone flap or especially difficult coagulation in the course of surgery [5,6]. Immediate diagnosis is important. Although the signs are not always revealing, the recurrent or progressive deterioration of the patient during or after the operation is quite suggestive, and BUS and CT scan are both effective examinations to reveal early EDH according to our experiences.

The EDH has been reported as intra-operative complication or to develop after the operation from 1 day to several days later, even months, but mainly within the 1^st^ week. Our research reviewed that the EDH developed within 5 days after the operation. In recent years the incidence is reducing with better understanding of the original disease and improvement of surgery skill. Yet when it happens it can still be fatal and requires urgent management, and it is also one of the major causes of urgent re-operation during the 1^st^ week after tumor resection. Prompt craniotomy surgery used to be recommended for abundant or continuous hemorrhage, sometimes with decompressive craniotomy for those with severe intracranial hypertension [7,8]. However, the craniotomy may cause further ICP fluctuation, forming an evil circle till out of control. Furthermore, for most urgent situations the craniotomy proves to be relatively time-wasting. According to our cases, EDH evacuation through craniotomy could result in recurrent regional EDH, regional cerebral hematoma, contra-lateral distant EDH, contra-lateral distant cerebral hematoma, or malignant intracranial hypertension, and 2 case of this study didn’t survive. It seems that both the complication rate and mortality rate of immediate re-operation increase significantly. The overall prognosis is poor, with prolonged hospitalization and heavier economic burden. A familiar condition is, distant epidural or intracranial hematoma keeps forming secondary to each operation, and with recurrent operation strike the patient fell. This compels us to investigate a more appropriate method of urgent management of the operation-related EDH [9].

The hemorrhage origin of EDH could be arterial, from the scalp, meningeal, or cerebral arteries, etc., or nonarterial, from the sinus, venous lakes, diploe, arachnoid granulations, etc. [10-12]. Continuous hemorrhage, ignoring the origin, splits the epidural space further on, which in turn aggravates the hemorrhage. The arterial or venous sinus hemorrhage requires more aggressive management and definite coagulation. Our new method does not suitable for these situations. However most atraumatic hemorrhage, especially operation-related EDH we discussed here, is from non-significant venous origin, as we discovered during re-operation of hematoma evacuation. This kind of hemorrhage is suggested to be stopped by compression hemostasis. Based on these entities: a. the intracranial epidural space is a potential space; b. the operation-related epidural hemorrhage is mostly from venous hemorrhage which can be stopped by compression hemostasis; c. most intra-operative adjacent or distant EDH may show specific signs, like protrusion of the cortex through a bone flap, difficulty in coagulation, or patients deterioration, and intra-operative BUS or CT scan can review definite EDH immediately and also measure the location and volume of the hemorrhage, we design this method of EDH management. First diagnose the EDH with BUS or CT immediately, than clear the hematoma with minimal invasive aspiration, and at last apply the vaccum drainage system into the epidural space. The purpose of this new method is to evacuate the existing hematoma through least invasive approach and to drain the further hemorrhage continuously while reconstruct the vacuum epidural space. With gelatin sponge insertion the vaccum also forms a forceful adhesion of the dura to the inner periosteum which stops the hemorrhage through compression hemostasis effectively. It’s prompted to mind of the possibility of further hemorrhage and the hemostasis impairment. To our experience this side-effect of vaccum drainage is not so severe that there’s no need of transfusion for the patient. While the continuous vacuum drainage is applied, the clinical state of the patient, the drainage volume, CBC, hemostasis function, and cranial CT scan are monitored daily. The drainage volume presents to reduce significantly in several days. The reduction of hematocrit and impairment of hemostasis are considered to be related to the blood loss during the operation of tumor resection. They are not quite significant and can be adjusted within several days. Daily CT scan reveals stable epidural space. The method appears to be as effective as craniotomy surgery on the extend of hematoma clearance. It is superior in urgent control of intra-operative epidural hemorrhage, easy application, less invasion, and low rate of complication. The vaccum drainage releases the compression caused by the hematoma continuously and gently with no significant secondary ICP fluctuation, and the patients tolerate better. Sure the overall prognosis is also affected by other factors like the entity of the primary tumor and the pre-operative clinical state of the patient, the operation-related EDH alone improves well after applying the vacuum drainage system. The disadvantage includes the risk of continuous blood loss during the early time. Close monitoring of patient and strong supportive treatment are highly recommended [13]. The vacuum epidural space reconstruction proves to be effective in compression hemostasis with concurrent supportive treatment. This can be verified through drainage volume document and CT scan, and these results also indicate the time of drainage removal. We adopt the drainage volume of less than 100 ml per day with no obvious epidural hyper-intensive signal on CT scan as the indication of stopping vacuum drainage. The residual drainage volume may be exudation and is not considered significant. Consistent epidural hyper-intensive signal on CT scan suggested coagulation of the hematoma, and urokinase irrigation can be helpful. The exact value of vacuum application is still under investigation, for excessive vacuum suction will aggregate further blood loss, and insufficient vacuum is not enough in preventing continuous hemorrhage. Moreover, for those with active epidural hemorrhage of arterial origin or so, the more invasive open surgery would still be necessary to achieve definite coagulation. On the other hand, coagulated epidural hematoma, which presents as consistent epidural hyper-intensive signal on CT scan, is also resistant to drainage method and requires hematoma evacuation through craniotomy. Compared to distal EDH, the adjacent EDH seems to be less affected by the disadvantages mentioned above, and the vaccum drainage method works more successful. The psychological burden with drainage is more acceptable compared to re-operation. No further complications related to epidural drainage have been noticed. During 4 to 7 months follow-up, no EDH recurrence or other complications are noticed either.

## Conclusions

Epidural space is a potential space which may be split during tumor resection by fluctuation of ICP. The traditional method of hematoma evacuation through craniotomy is time-wasting and has a high incidence of complication. The new method of evacuating the existing hematoma through least invasive approach and draining the further hemorrhage while reconstruct the vacuum epidural space through vacuum drainage present to be a promising method to control operation-related EDH, especially superior in intra-operative adjacent EDH management. The advantages include immediate effectiveness, easy application, little complication, well-toleration, and satisfactory prognosis. More details including monitor method and some critical values remain to be standardized.

## Abbreviations

EDH: Epidural hematoma; ICP: Intracranial pressure; CSF: Cerebral spinal fluid; BUS: B-ultrasound; CT: Computed tomography; CBC: Complete blood count.

## Competing interests

The authors report no financial or other conflicts of interests concerning the materials or methods used in this study or the findings specified in this paper.

## Authors’ contributions

HL participated in the design of the study and performed the results analysis. LC carried out the ultrasound studies, participated in the operations and analyzed the ultrasound images. SL conceived of the study, and participated in its design and coordination and helped to draft the manuscript. All authors read and approved the final manuscript.
